# Instability of the mitochondrial alanyl-tRNA synthetase underlies fatal infantile-onset cardiomyopathy

**DOI:** 10.1093/hmg/ddy294

**Published:** 2018-10-04

**Authors:** Ewen W Sommerville, Xiao-Long Zhou, Monika Oláhová, Janda Jenkins, Liliya Euro, Svetlana Konovalova, Taru Hilander, Angela Pyle, Langping He, Sultan Habeebu, Carol Saunders, Anna Kelsey, Andrew A M Morris, Robert McFarland, Anu Suomalainen, Gráinne S Gorman, En-Duo Wang, Isabelle Thiffault, Henna Tyynismaa, Robert W Taylor

**Affiliations:** 1 Wellcome Centre for Mitochondrial Research, Institute of Neuroscience, Newcastle University, Newcastle upon Tyne, UK; 2 State Key Laboratory of Molecular Biology, CAS Center for Excellence in Molecular Cell Science, Shanghai Institute of Biochemistry and Cell Biology, Chinese Academy of Sciences, University of Chinese Academy of Sciences, Shanghai, China; 3 Center for Pediatric Genomic Medicine, Children's Mercy Hospital, Kansas City, MO, USA; 4 Research Programs Unit, Molecular Neurology, University of Helsinki, Helsinki, Finland; 5 Department of Pathology and Laboratory Medicine, Children's Mercy Hospital, Kansas City, MO, USA; 6 School of Medicine, University of Missouri Kansas City, Kansas City, MO , USA; 7 Institute of Human Development, University of Manchester, Manchester M13 9PL, UK; Willink Metabolic Unit, Genomic Medicine, Saint Mary’s Hospital, Manchester University Hospitals NHS Foundation Trust, Manchester M13 9WL, UK; 8 Neuroscience Center, Helsinki Institute of Life Sciences, University of Helsinki, Helsinki Finland; 9 Department of Neurosciences, Helsinki University Hospital, Helsinki, Finland

## Abstract

Recessively inherited variants in *AARS2* (NM_020745.2) encoding mitochondrial alanyl-tRNA synthetase (mt-AlaRS) were first described in patients presenting with fatal infantile cardiomyopathy and multiple oxidative phosphorylation defects. To date, all described patients with *AARS2*-related fatal infantile cardiomyopathy are united by either a homozygous or compound heterozygous c.1774C>T (p.Arg592Trp) missense founder mutation that is absent in patients with other *AARS2*-related phenotypes. We describe the clinical, biochemical and molecular investigations of two unrelated boys presenting with fatal infantile cardiomyopathy, lactic acidosis and respiratory failure. Oxidative histochemistry showed cytochrome *c* oxidase-deficient fibres in skeletal and cardiac muscle. Biochemical studies showed markedly decreased activities of mitochondrial respiratory chain complexes I and IV with a mild decrease of complex III activity in skeletal and cardiac muscle. Using next-generation sequencing, we identified a c.1738C>T (p.Arg580Trp) *AARS2* variant shared by both patients that was *in trans* with a loss-of-function heterozygous *AARS2* variant; a c.1008dupT (p.Asp337*) nonsense variant or an intragenic deletion encompassing *AARS2* exons 5–7. Interestingly, our patients did not harbour the p.Arg592Trp *AARS2* founder mutation. *In silico* modelling of the p.Arg580Trp substitution suggested a deleterious impact on protein stability and folding. We confirmed markedly decreased mt-AlaRS protein levels in patient fibroblasts, skeletal and cardiac muscle, although mitochondrial protein synthesis defects were confined to skeletal and cardiac muscle. *In vitro* data showed that the p.Arg580Trp variant had a minimal effect on activation, aminoacylation or misaminoacylation activities relative to wild-type mt-AlaRS, demonstrating that instability of mt-AlaRS is the biological mechanism underlying the fatal cardiomyopathy phenotype in our patients.

## Introduction

Mitochondrial respiratory chain disorders are among the most common early onset metabolic disorders with an estimated minimum prevalence of 1 in 5000 live births (1). Isolated or multiple deficiencies of the five multimeric complexes (I–V) that comprise the oxidative phosphorylation (OXPHOS) system are associated with broad clinical, biochemical and genetic heterogeneity. Disorders of mitochondrial mRNA translation or protein synthesis are especially important causes of multiple mitochondrial respiratory chain deficiency, which are linked to both mitochondrial DNA (mtDNA) and nuclear gene defects (2).

Following post-transcriptional modification, a critical step of mitochondrial protein synthesis is the aminoacylation or ‘charging’ of transfer RNAs (tRNAs) (3). This step involves the recognition and conjugation of amino acids with their corresponding cognate mitochondrial transfer RNA (mt-tRNA), as dictated by the codon sequence. Attachment is catalysed by mitochondrial aminoacyl-tRNA synthetases (mt-aaRS) that are encoded by nuclear genes and imported into mitochondria. There are 17 mt-aaRS and two dual cytosolic-mitochondrial synthetases (GlyRS, LysRS), while mt-GluRS is required to efficiently misaminoacylate tRNA^Gln^ to form Glu-tRNA^Gln^ in mitochondria (4,5).

All mt-aaRS and dual-localized synthetases are associated with autosomal recessive human disorders manifesting in clinically and biochemically heterogeneous phenotypes (6–8). Despite ubiquitous expression, autosomal recessive mt-aaRS disorders are associated with intriguing tissue- and cell-specific phenotypes that typically involve the central nervous system (9–18). High-throughput, next-generation sequencing technologies have greatly expanded the phenotypic continuum of mt-aaRS disorders to encompass patients presenting with additional clinical features or with the absence previously considered salient features.

Recessively inherited variants in *AARS2* (NM_020745.2), encoding mitochondrial alanyl-tRNA synthetase (mt-AlaRS), were first described in patients presenting with fatal infantile cardiomyopathy and multiple OXPHOS defects (19), with additional patients subsequently identified (20–24). However, the spectrum of *AARS2*-related disease has expanded to include childhood to adulthood-onset leukoencephalopathy with premature ovarian failure (POF) in females (9,25–28), retinopathy and optic atrophy (29) and fatal non-immune hydrops fetalis (30); all with conspicuous absence of cardiac involvement. Currently, *AARS2*-related fatal infantile cardiomyopathy is associated with a recurrent pathogenic c.1774C>T (p.Arg592Trp) founder mutation that is either homozygous or compound heterozygous in all described patients. This founder mutation has not been reported in patients presenting with other *AARS2-*related phenotypes. Consequently, the spectrum of *AARS2*-related disease phenotypes has been attributed to the location of pathogenic variants in the protein and the effect on protein function (22). It has been previously hypothesized that the p.Arg592Trp *AARS2* founder mutation, which occurs in a conserved editing domain, causes a severe decrease in aminoacylation due to impaired tRNA binding and positioning of the 3′-end within the active site (22). On the other hand, other *AARS2*-related disease phenotypes were predicted to result from only a partial reduction in aminoacylation activities (22). This mt-AlaRS editing domain is required for the deacylation of mischarged tRNAs, since the aminoacylation domain is unable to discriminate alanine with serine and glycine (31,32). This proofreading activity is essential to clear mischarged Ser-tRNA^Ala^ and avoid misincorporation of serine at alanine codons, since a slight decrease results in embryonic lethality in mice (33). Of all mt-aaRS, only mt-AlaRS and mt-ThrRS have demonstrable editing activities to prevent the formation of mischarged mt-tRNAs (31,32,34).

In this study, we describe two unrelated patients presenting with fatal infantile cardiomyopathy, lactic acidosis and respiratory failure, with severe multiple OXPHOS deficiency and who both harboured an unreported *AARS2* variant (c.1738C>T, p.Arg580Trp) *in trans* with a loss-of-function *AARS2* variant, but not the recurrent p.Arg592Trp founder mutation. We validate pathogenicity of this shared novel mt-AlaRS editing domain variant through post-mortem molecular studies, *in silico* modelling and *in vitro* assays. This data supports the genotype–phenotype correlation between *AARS2* variants in the ß-barrel domain with fatal cardiomyopathy and that instability of mt-AlaRS due to the novel p.Arg592Trp and loss-of-function alleles is the underlying biological mechanism in our patients.

## Results

### Case reports

#### Patient 1

Patient 1 was a male infant born at term by normal vaginal delivery to non-consanguineous parents with a birth weight of 3.11 kg. He developed respiratory distress and poor respiratory drive soon after birth, requiring ventilation. He had generalized hypotonia and evidence of diaphragmatic paralysis with paradoxical abdominal wall movements. There was persistent lactic acidemia (9–30 mmol/L; normal <2.5 mmol/L). Urine organic and amino acids were unremarkable apart from increased lactate excretion. There were no seizures and he tolerated nasogastric feeding. Initial echocardiography showed no evidence of cardiomyopathy, but he developed mild biventricular hypertrophy by 5 weeks of age. This was associated with periods of cardiac electrical inactivity, lasting 6–7 s and later up to 30 s. He was weaned off ventilatory support at 6 weeks and died 1 week later. Whole mitochondrial genome sequencing failed to detect a pathogenic variant, while quantitative real-time polymerase chain reaction (PCR) assay of skeletal muscle mtDNA copy number was normal (data not shown). A previous daughter was born at term and died within 24 h with lactic acidemia and coagulopathy. Post-mortem analysis of this female sibling is said to have shown pulmonary hypoplasia. There is one healthy son and the child’s mother had two previous miscarriages.

#### Patient 2

**Figure 1:**
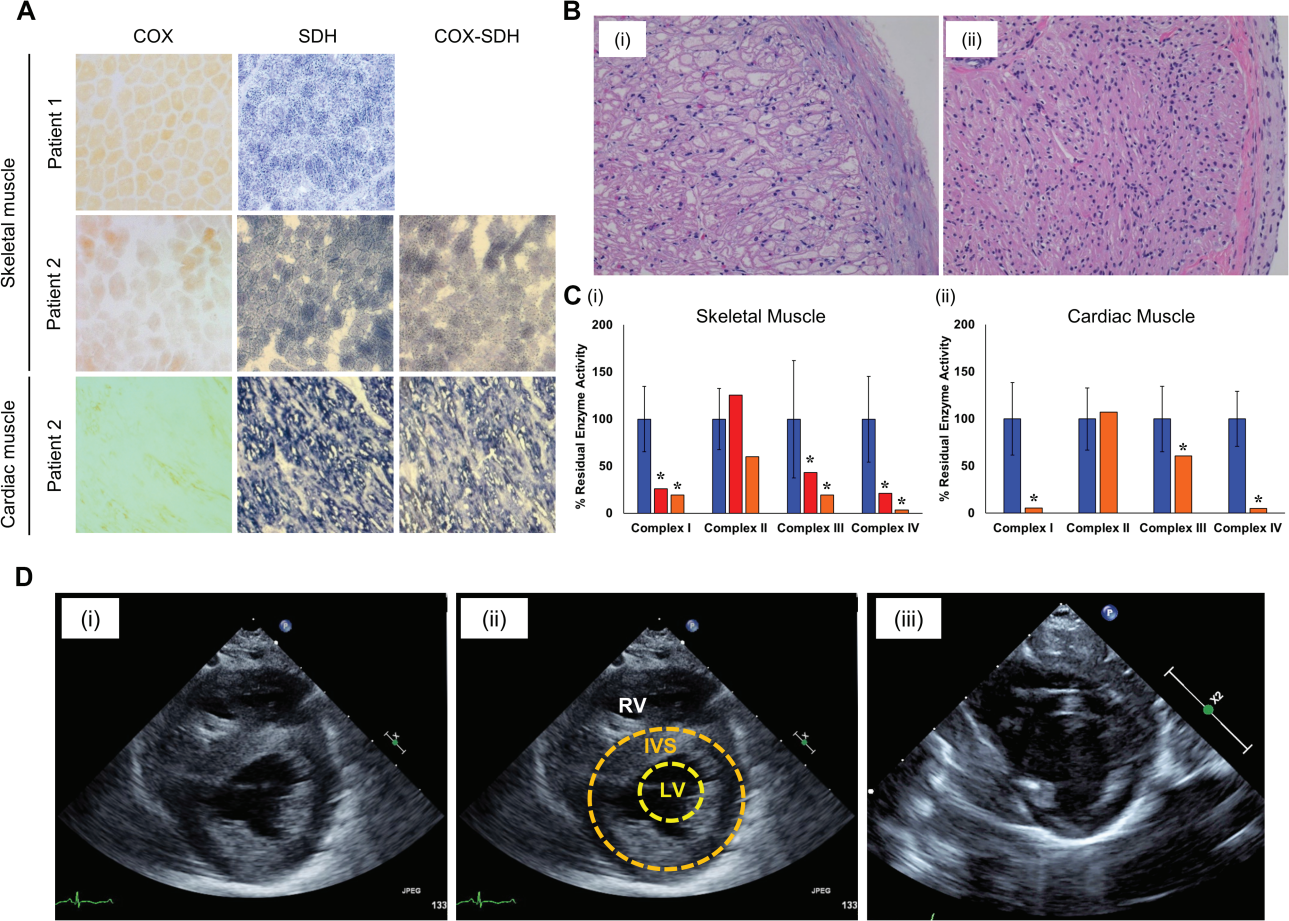
Histochemical and biochemical studies of *AARS2* patient skeletal and cardiac muscle. (**A**) Diagnostic skeletal and cardiac muscle were subjected to COX, SDH and sequential COX-SDH histochemical reactions. Skeletal muscle from Patient 1 was not subjected to sequential COX-SDH histochemistry. (**B**) Photomicrograph of (i) Patient 2 cardiac muscle sampled at autopsy and (ii) healthy heart of a child of similar age. (**C**) Measurement of mitochondrial OXPHOS activities (CI-CV) normalized to CS in skeletal (Patient 1 and 2) and cardiac muscle (Patient 2), as a percentage of residual controls. Controls are denoted in blue, Patient 1 in red and Patient 2 in orange. Decreased OXPHOS activities are denoted by asterisks (‘*’). (**D**) Echocardiographic images in the parasternal short axis view of Patient 2’s heart (i and ii; annotated in panel ii) and the normal heart of a child approximately the same age (iii). The patient’s heart demonstrates severe concentric hypertrophy of the left ventricle (LV) involving both the interventricular septum (IVS) and the posterior wall. Also identified is the right ventricle (RV).

Patient 2, a male infant, was a dizyotic twin born at 33 weeks gestation by caesarean section to non-consanguineous parents with a birth weight of 1.595 kg. He presented at 2 months of age with respiratory failure secondary to respiratory syncytial virus bronchiolitis. An echocardiogram revealed severe concentric left ventricular hypertrophy and dilation and severe systolic dysfunction. (Fig. 1D) Brain magnetic resonance imaging noted a thin corpus callosum, but neurological examination was normal. He required ongoing mechanical ventilation for respiratory failure. He received two courses of venoarterial extracorporeal membrane oxygenation for circulatory support and was transitioned to a Berlin left ventricular assist device at 5 months of age. Aside from persistent lactic acidemia (2–16 mmol/L; normal <2.5 mmol/L), extensive biochemical evaluation was unremarkable. He developed multi-organ failure and suffered a left middle cerebral artery stroke with residual neurologic dysfunction and muscular weakness at 6 months of age. Palliative care was initiated and he died at 7 months of age. Post-mortem evaluation demonstrated a markedly enlarged globular heart with biventricular hypertrophy and severe myocyte vacuolization. Negative genetic evaluations included karyotype, single nucleotide polymorphism (SNP) microarray, urine mitochondrial genome analysis and a targeted 89-gene cardiomyopathy panel. The patients’ male twin was diagnosed with Trisomy 18 prenatally and died at birth with unknown cardiac status. An older female sibling was born at term and died at birth with significant cardiomegaly noted post-mortem. Parental echocardiograms were normal.

### Diagnostic histochemical and biochemical analyses of skeletal and cardiac muscle reveal severe multiple mitochondrial OXPHOS defects

Histopathologic analysis of skeletal muscle from Patient 1 showed vacuolated fibres with increased lipid. Oxidative enzyme histochemistry showed absent *c* oxidase (COX) activity in ~50% of fibres (Fig. 1A), although an assessment of sequential COX–succinate dehydrogenase (SDH) histochemistry was not made. In Patient 2, oxidative enzyme histochemistry of post-mortem skeletal and cardiac muscle revealed global COX-deficiency (Fig. 1A). Post-mortem histopathologic analysis of the heart noted marked biventricular myocyte vaculosation, myocyte hypertrophy and mild subendocardial fibrosis (Fig. **1**Bi), compared to an age-matched control (Fig. **1**Bii). Electron microscopy of cardiac muscle showed no ultrastructural evidence for a mitochondrial disorder but demonstrated vacuolar myopathic changes with large membrane bound vesicles containing glycogen.

Biochemical analysis of mitochondrial respiratory chain complex activities (Fig. 1C) revealed markedly decreased complex I and complex IV activities with low complex III activity in Patient 1 and Patient 2 skeletal muscle relative to controls. Similarly, severe complex I and complex IV activities with low complex III activity were also noted in the cardiac muscle from Patient 2, relative to age-matched controls.

### Identification of recessively inherited AARS2 variants

**Figure 2:**
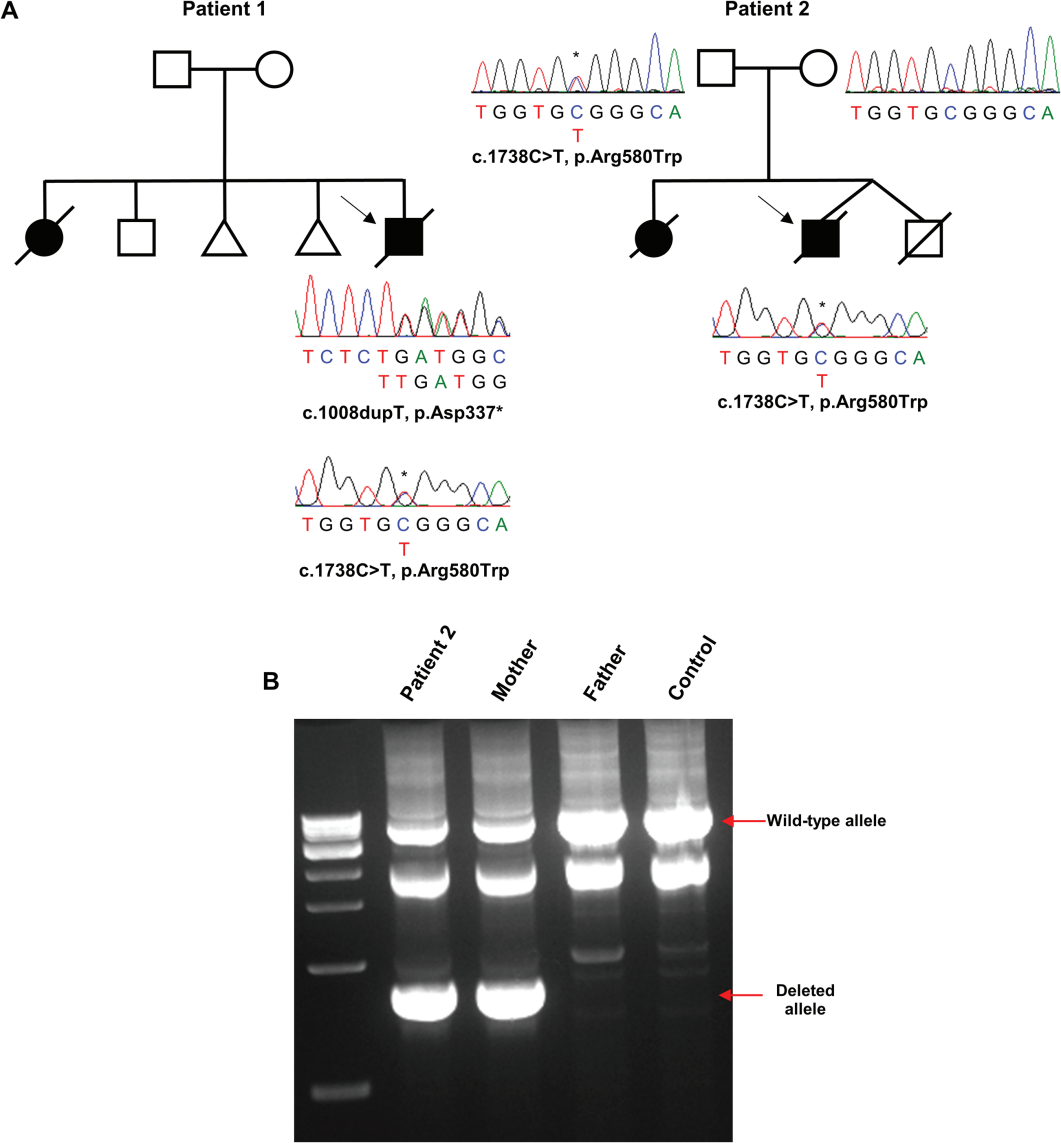
Genetic analysis of identified *AARS2* variants. (**A**) Family pedigrees showing Sanger sequencing confirmation of the c.1008dupT (p.Asp337*) and c.1738C>T (p.Arg580Trp) *AARS2* variants for Patient 1 and segregation of the c.1738C>T (p.Arg580Trp) variant for Patient 2. (**B**) Long-range PCR confirmation of a maternally inherited, heterozygous intragenic 4.1 kb deletion on the short arm of chromosome 6p21.1 encompassing exons 5–7 of *AARS2*. A non-specific product at ~2 kb does not affect segregation analysis. The wild-type allele (5 kb) and the deleted allele (1 kb) are denoted by a solid red arrow.

In Patient 1, analysis of whole exome sequencing (WES) called variants in nuclear genes encoding mitochondrial-localized proteins revealed two heterozygous variants in *AARS2* (NM_020745.2); c.1008dupT, (p.Asp337*) and c.1738C>T (p.Arg580Trp), which were confirmed by Sanger sequencing (Fig. 2A). Unfortunately, familial segregation studies were not possible. In Patient 2, whole genome sequencing (WGS) revealed the identical heterozygous c.1738C>T (p.Arg580Trp) *AARS2* missense variant that was paternally inherited and confirmed by Sanger sequencing (Fig. 2A). Visual inspection of read alignments and using IGV (35) also identified a maternally inherited, intragenic 4.1 kb deletion on the short arm of chromosome 6p21.1 encompassing exons 5–7 of *AARS2*, which was confirmed to segregate with the disease by long-range PCR (Fig. 2B).

None of the identified variants were previously reported as pathogenic and both patients did not harbour the p.Arg592Trp *AARS2* founder mutation. In GnomAD, the c.1008dupT(p.Asp337*) variant was present in 3/245814 (minor allele frequency (MAF) = 1.22 × 10^−5^) alleles and the c.1738C>T (p.Arg580Trp) variant was present in 9/246174 (MAF = 3.656 × 10^−5^) alleles, all in heterozygous state. The p.Arg580Trp variant was predicted to be damaging by the *in silico* tools PolyPhen-2 (HumDiv Score 0.996) (36) and SIFT (Score 0.02) (37) but was predicted to be benign by Align GVGD (Class 0, GV: 127.27, GD: 46.61) (38).

According to the American College of Medical Genetics guidelines for characterization of sequence variants, the novel *AARS2* p.Arg580Trp variant did not meet the criteria for classification as ‘likely pathogenic’ variant. Therefore, we decided to pursue functional analyses to confirm pathogenicity of the identified *AARS2* variants.

### Structural modelling of the p.Arg580Trp AARS2 variant

**Figure 3:**
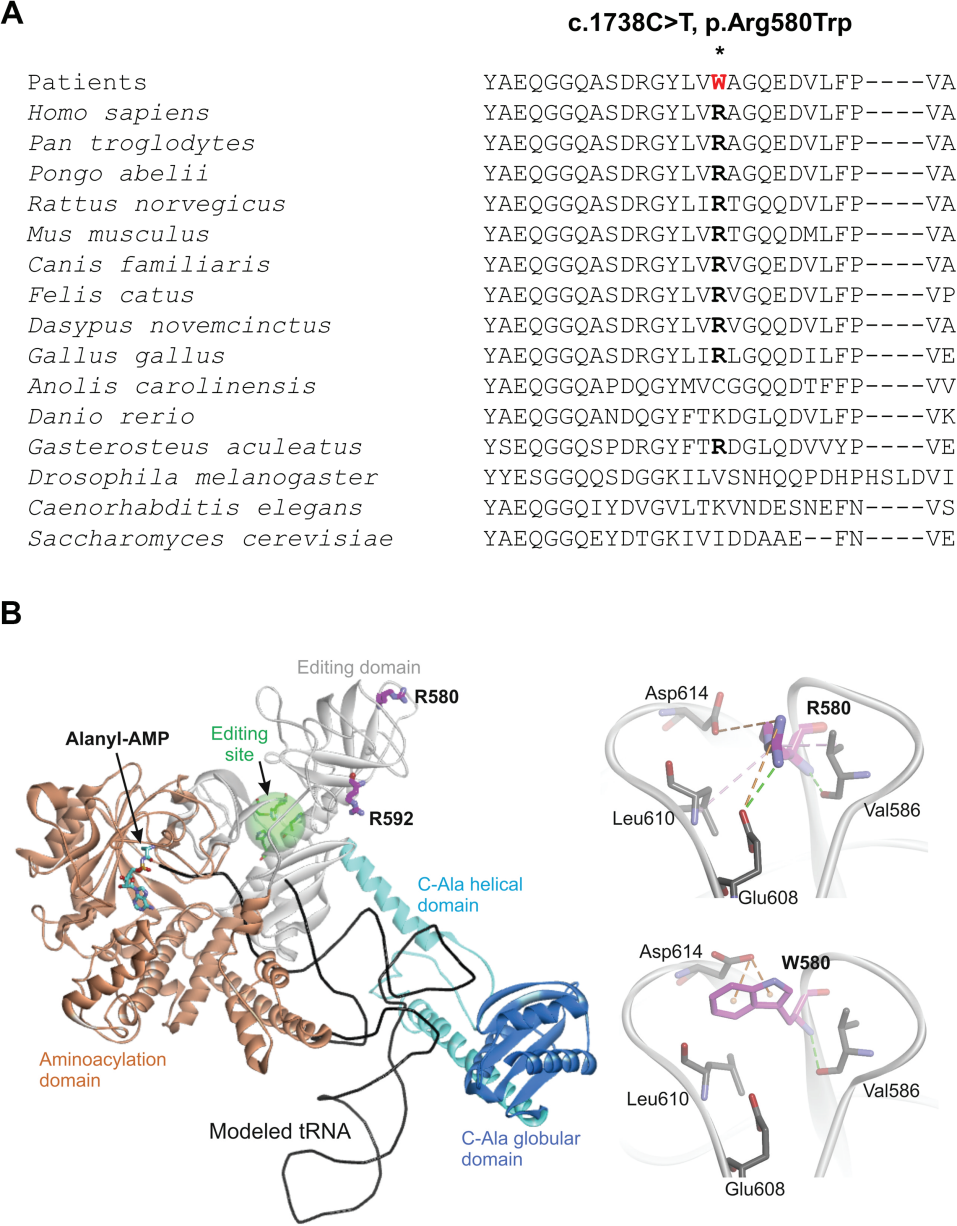
*In silico* modelling of the human mt-AlaRS Arg580 residue and p.Arg580Trp variant. (**A**) Multiple sequence alignment of the mt-AlaRS Arg580 residue across species. (**B**) *In silico* modelling of the p.Arg580Trp variant on mt-AlaRS protein stability and intramolecular bonds. Editing site is highlighted with transparent green sphere. It contains zinc-binding residues His632, His636, His753 and Cys749.

We first examined the sequence conservation of the Arg580 residue and modelled the p.Arg580Trp variant using the available structural model for mt-AlaRS (22). The Arg580 residue is conserved in this position among mt-AlaRS in mammals and birds but not in the lizard *Anolis carolinensis*, fish *Danio rerio*, fly *Drosophila melanogaster*, worm *Caenorhabditis elegans* or yeast *Saccharomyces cerevisiae* (Fig. 3A). However, a bulky aromatic residue in the corresponding position is not found in any of the analysed mt-AlaRS sequences. Arg580 is one of the solvent exposed residues on the surface of the β-barrel subdomain (530–621 aa) of the mt-AlaRS editing domain and is involved in complex electrostatic, hydrophobic and hydrogen interactions with neighbouring residues. This suggests a structural role for Arg580 and an impact on protein folding and stability. Substitution of an arginine to a hydrophobic and bulky tryptophan is predicted to affect folding of the β-barrel subdomain and as a result compromise the stability of the entire protein (Fig. 3B).

### mt-AlaRS protein levels are diminished in patient fibroblasts without defective mitochondrial protein synthesis

**Figure 4:**
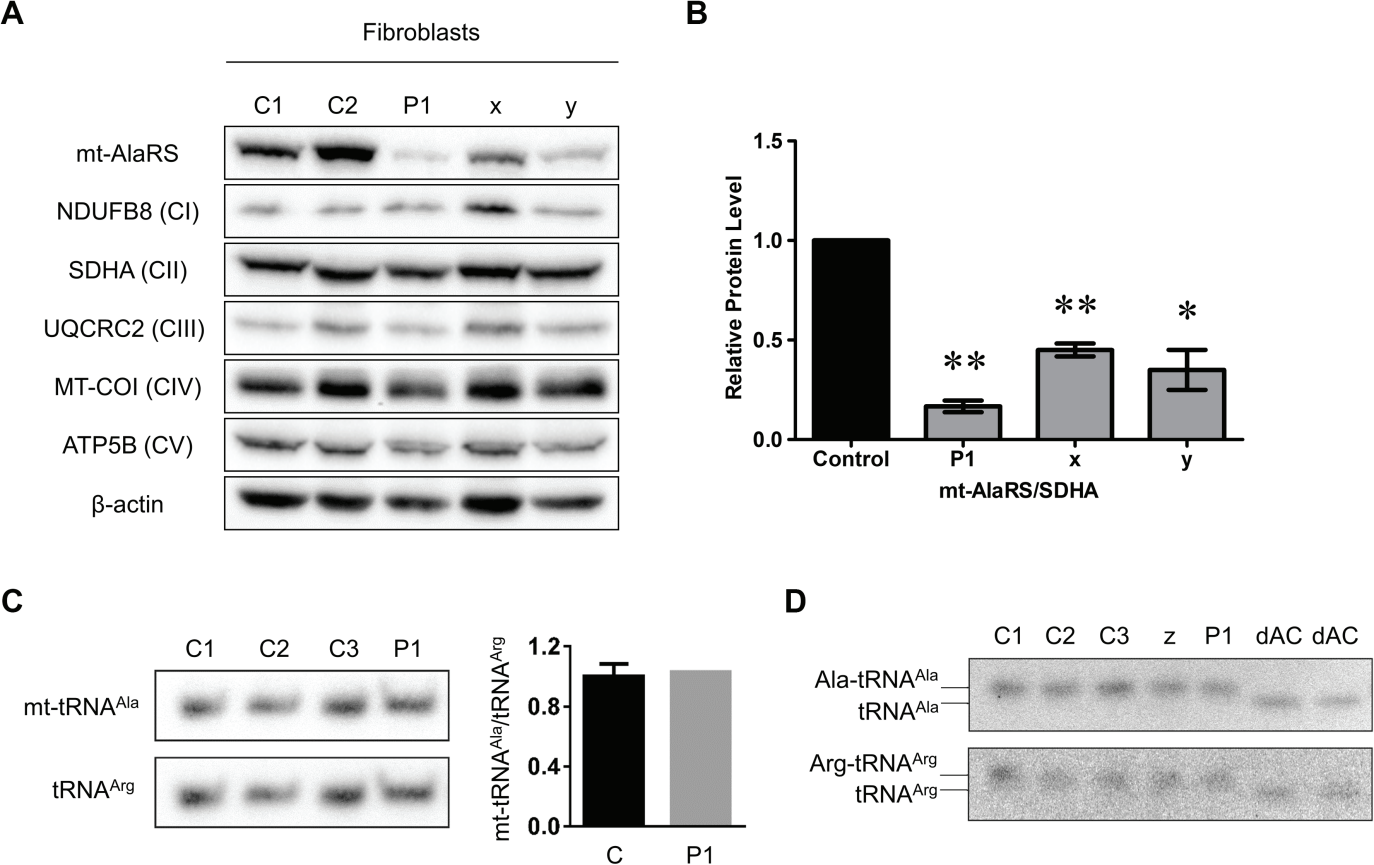
Western blot, northern blot and aminoacylation analysis in *AARS2* patient fibroblast lysates. (**A**) Steady-state mt-AlaRS and OXPHOS subunit protein levels in Patient 1 (P1) and two control fibroblast lysates. Also shown are two reported *AARS2* patients in the literature who were homozygous or compound heterozygous for the p.Arg592Trp founder mutation; Patient x (homozygous p.Arg592Trp) corresponds to Patient 11, and Patient y (p.Arg592Trp and c.2882C>T (p.Ala961Val)) corresponds to Patient 7 in (24). Antibodies against mt-AlaRS, NDUFB8 (CI), SDHA (CII), UQCRC2 (CIII), MT-COI (CIV) and ATP5B (CV) were used, with β-actin as a loading control. (**B**) Graph of relative mt-AlaRS protein levels (n = 3) in controls, Patient 1 (P1), Patient x (x) and Patient y (y) fibroblasts. All data were normalized to SDHA and represented as mean ± standard error of the mean. Significant difference between controls and patient fibroblasts is indicated by asterisks above the columns (*, *P*-value < 0.05; **, *P*-value < 0.01 by two-tailed paired students *t*-test). (**C**) Northern blot analysis of mt-tRNA^Ala^ levels in patient fibroblasts. (**D**) Aminoacylation assay showing aminoacylated (‘charged’) and deacylated (‘uncharged’) mt-tRNA^Ala^ in patient fibroblasts. Mitochondrial tRNA^Arg^ was used as a loading control. Lower bands in the dAc lanes denote fully deacylated control tRNA species. Patient z was homozygous for the p.Arg592Trp founder mutation and has been previously reported, corresponding to Patient 1 in (19).

We assessed steady-state mt-AlaRS and OXPHOS complex subunit protein levels in fibroblast lysates from Patient 1 and two patients harbouring the p.Arg592Trp *AARS2* founder mutation on at least one allele (Fig. 4A). Cultured fibroblasts were not available from Patient 2. Quantification of steady-state levels of mt-AlaRS showed a statistically significant decrease in all patients (Fig. 4B). However, there was no change in OXPHOS complex subunit levels (Fig. 4A). Levels of mt-tRNA^Ala^ and the presence of uncharged and charged species were also assessed in patient fibroblasts. Northern blot analysis of total RNA from fibroblasts of Patient 1 and an unrelated patient who was homozygous for the recurrent p.Arg592Trp founder mutation showed no change in the abundance of uncharged mt-tRNA^Ala^ (Fig. 4C). Analysis of aminoacylated mt-tRNA^Ala^ showed the presence of charged Ala-tRNA^Ala^ species with no uncharged tRNA^Ala^ in both patient and control fibroblasts (Fig. 4D).

### Patient skeletal and cardiac muscle have decreased mt-AlaRS protein levels and mitochondrial protein synthesis defects

**Figure 5:**
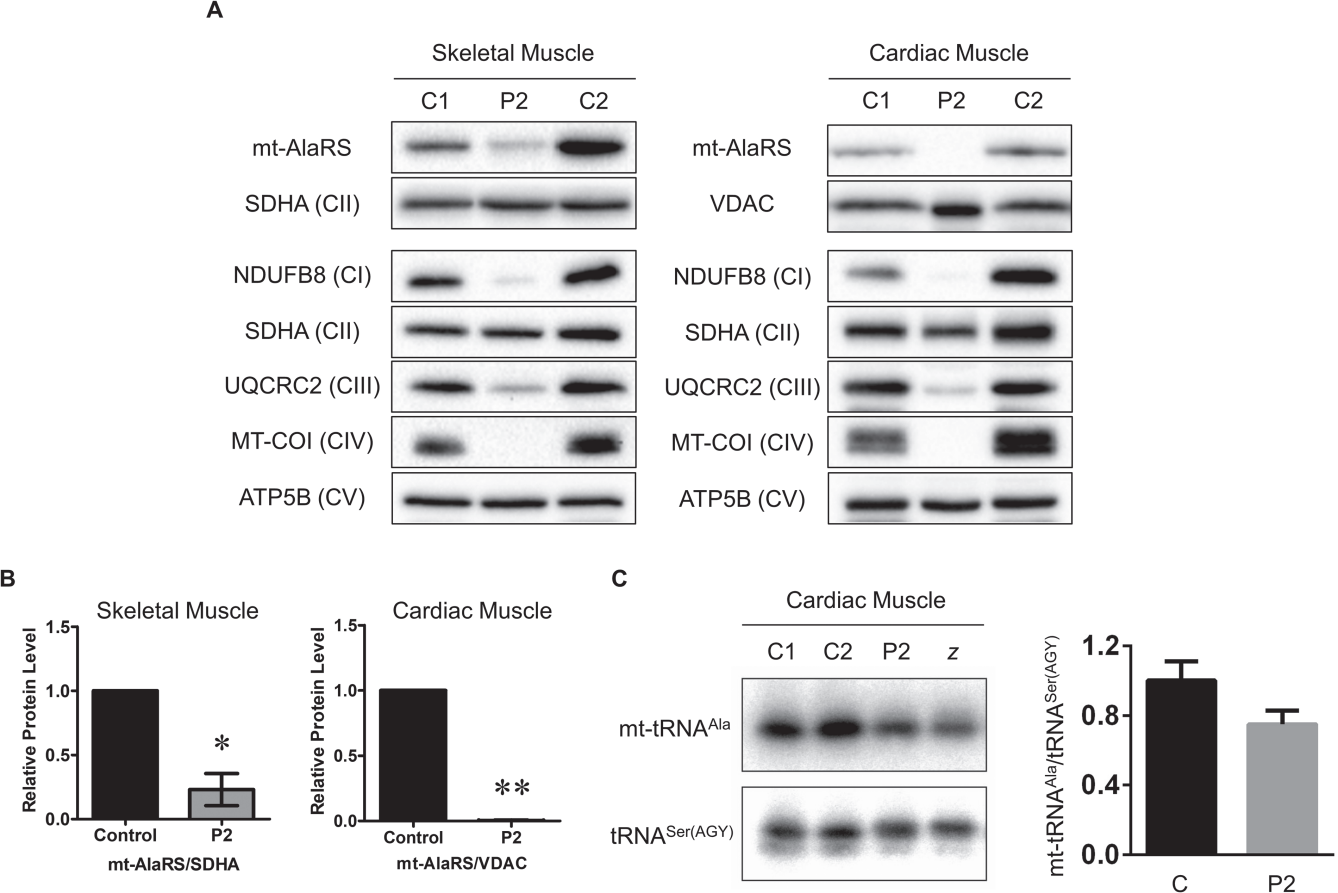
Western blot and northern blot analysis in *AARS2* patient skeletal and cardiac muscle homogenates. (**A**) Steady-state mt-AlaRS and OXPHOS subunit protein levels in Patient 2 skeletal and cardiac muscle homogenates. Antibodies against mt-AlaRS, NDUFB8 (CI), SDHA (CII), UQCRC2 (CIII), MT-COI (CIV) and ATP5B (CV) were used, with SDHA as a loading control. (**B**) Graphs of relative mt-AlaRS protein levels in control and Patient 2 (P2) skeletal (n = 3) and cardiac muscle (n = 2) homogenate. All data were normalized to SDHA (skeletal muscle) or VDAC (cardiac muscle) and represented as mean ± standard error of the mean. Significant difference between controls and patient homogenates is indicated by asterisks above the columns (*, *P*-value < 0.05; **, *P*-value < 0.01 by two-tailed paired students *t*-test). (**C**) Northern blot analysis of mt-tRNA^Ala^ levels in Patient 2 and Patient z who was homozygous for the p.Arg592Trp founder mutation, corresponding to Patient 1 in (19). Mitochondrial tRNA^Ser(AGY)^ was used as a loading control.

Next, we examined steady-state mt-AlaRS and OXPHOS subunit protein levels in skeletal and cardiac muscle homogenates from Patient 2 (Fig. 5A). No skeletal or cardiac muscle was available from Patient 1. Quantification of steady-state mt-AlaRS protein levels showed a statistically significant decrease in both skeletal and cardiac muscle homogenate from Patient 2; mt-AlaRS levels were undetectable in cardiac homogenate (Fig. 5B). There was marked loss of MT-COI (complex IV) and NDUFB8 (complex I) subunits with a mild reduction of UQCRC2 (complex III). This was consistent with the decreased biochemical activities for complexes I, III and IV in skeletal and cardiac muscle (Fig. 1C). We also quantified *AARS2* mRNA levels in cardiac muscle, which confirmed an ~50% decrease in *AARS2* mRNA relative to controls (data not shown). We then assessed mt-tRNA^Ala^ levels in cardiac muscle from Patient 2 as well as an unrelated patient who was homozygous for the p.Arg592Trp founder mutation. Northern blot analysis showed decreased levels of uncharged mt-tRNA^Ala^ in both patients (Fig. 5C). Unfortunately, the presence of charged and uncharged mt-tRNA^Ala^ could not be assessed.

### The p.Arg580Trp AARS2 variant likely has a minimal effect on mitochondrial protein synthesis

**Figure 6:**
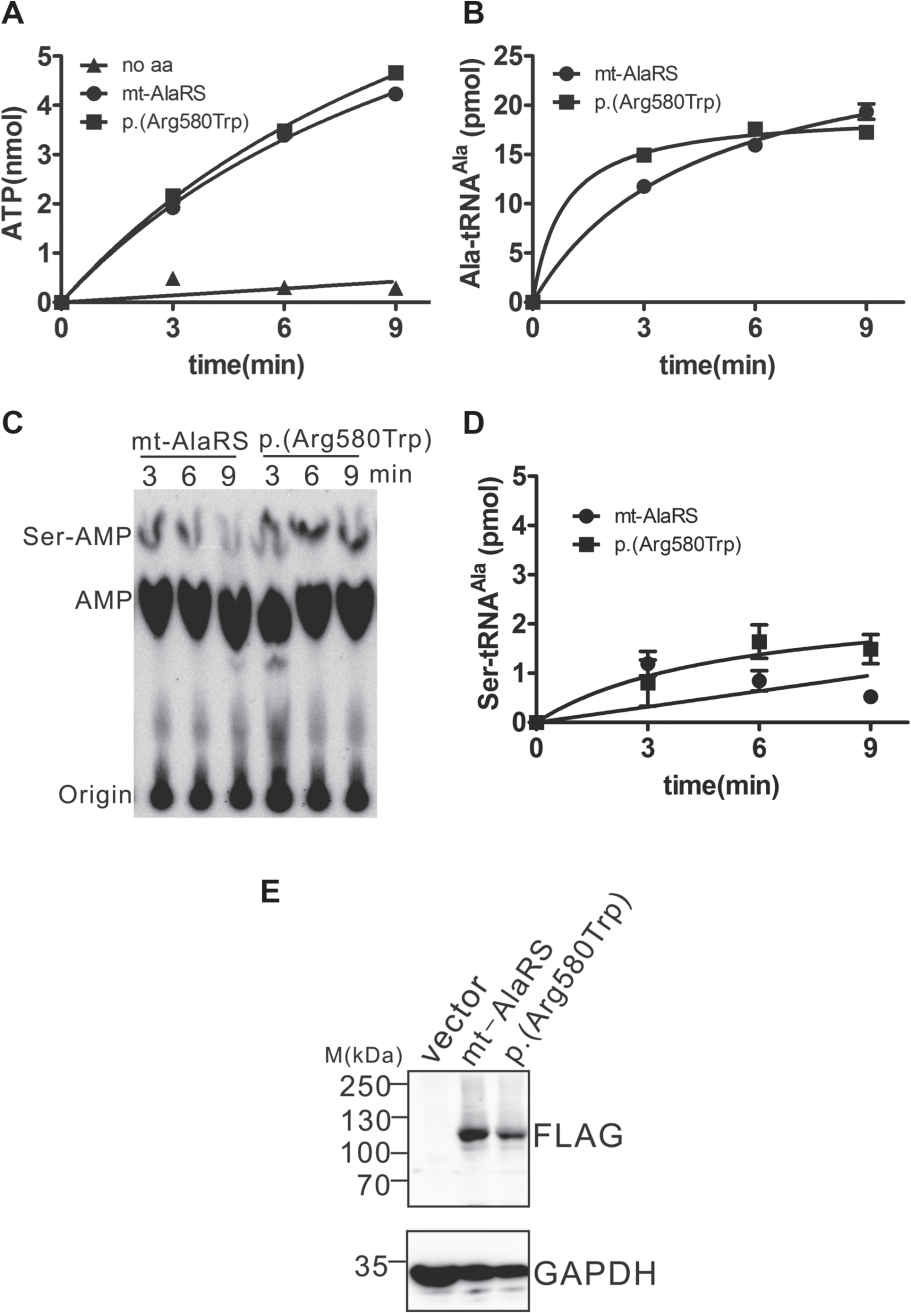
*In vitro* studies of the human mt-AlaRS p.Arg580Trp mutant. (**A**) ATP-PPi exchange determination of human mt-AlaRS (•) and the p.Arg580Trp mutant (▪). A reaction at the absence of Ala was included for a control (▴). (**B**) Aminoacylation activity of human mt-AlaRS (•) and the p.Arg580Trp mutant (▪). (**C**) A representative TLC showing the mischarging of mt-tRNA^Ala^ by human mt-AlaRS and the p.Arg580Trp mutant. Nuclease S1-generated Ser-[^32^P]AMP (reflecting Ser-[^32^P]tRNA^Ala^) and [^32^P]AMP (reflecting free [^32^P]tRNA^Ala^) were separated by TLC. (**D**) Graph of the mischarging activity of human mt-AlaRS (•) and the p.Arg580Trp mutant (▪). In all graphs, the data represent the mean values with error bars indicating standard deviation (SD). (**E**) Steady-state protein level of overexpressed human mt-AlaRS and the p.Arg580Trp mutant in HEK293T cells. Genes encoding C-terminal FLAG-tagged human mt-AlaRS and p.Arg580Trp mutant were overexpressed in HEK293T cells and the proteins were detected by FLAG antibodies. GAPDH was detected as a loading control.

We assessed the aminoacylation and editing activities of mt-AlaRS with the p.Arg580Trp variant *in vitro*. ATP-PPi exchange reaction assay showed that the p.Arg580Trp variant exhibited the same amino acid activation activity compared to wild-type mt-AlaRS, suggesting that there is no direct impact on the synthetic active site (Fig. 6A). Next, an aminoacylation assay showed that the p.Arg580Trp variant had comparable tRNA^Ala^ charging activity compared to wild-type human mt-AlaRS, suggesting that the speed of mitochondrial protein synthesis was not affected (Fig. 6B).

To more accurately determine any defect in translational quality control, [^32^P]tRNA^Ala^ was used in a misaminoacylation assay. The free [^32^P]tRNA^Ala^ and the mischarged Ser-[^32^P]tRNA^Ala^ were hydrolysed by nuclease S1 and the produced [^32^P]AMP and Ser-[^32^P]AMP could be directly observed (Fig. 6C and D). The p.Arg580Trp variant produced only negligibly (if any) more Ser-tRNA^Ala^ when compared to wild-type human mt-AlaRS, suggesting fidelity of protein synthesis was not significantly impacted.

Finally, we overexpressed wild-type human mt-AlaRS and the p.Arg580Trp mutant in HEK293T cells, demonstrating that steady-state levels of the mt-AlaRS p.Arg580Trp mutant were decreased relative to wild-type (Fig. 6E), which was consistent with *in silico* modelling and decreased levels in patient tissues.

## Discussion

Autosomal recessive *AARS2* variants were first described in patients with fatal infantile cardiomyopathy (19). Recently, the clinical spectrum has expanded to include childhood and adult-onset leukodystrophy (with POF in females) (9), retinopathy and optic atrophy (29) and fatal non-immune hydrops fetalis (30). However, cardiomyopathy is conspicuously absent in more recently described phenotypes. Currently, there are 14 reported patients presenting with *AARS2*-related fatal infantile cardiomyopathy (19,20,22–24) and one patient who died *in utero* with myopathy, hypotonia and multiple fractures (21). These reported patients all harbour at least one copy of the recurrent p.Arg592Trp missense founder mutation.

In contrast, we report two unrelated patients presenting with fatal infantile cardiomyopathy, lactic acidosis and respiratory failure, with severe multiple OXPHOS defects and who harboured biallelic *AARS2* variants but not the recurrent founder allele. Instead, our patients both harboured a novel p.Arg580Trp missense variant that was compound heterozygous with a second, loss-of-function *AARS2* variant. Nonetheless, the clinical presentation of our patients was severe and broadly comparable to the previously reported patients harbouring the p.Arg592Trp founder mutation on at least one allele. For all patients, the clinical course was fatal before 1 year of life with onset of cardiac features either before or shortly after birth.

Mt-AlaRS is one of two reported mitochondrial synthetases that have editing activities to prevent the formation of mischarged mt-tRNAs (31,32,34). The conserved editing domain of mt-AlaRS is essential since the aminoacylation domain cannot discriminate alanine with serine and glycine, thus avoiding the misincorporation of serine at alanine codons and clearing mischarged Ser-tRNA^Ala^ (31,32). Both p.Arg580Trp and p.Arg592Trp variants are located in the β-barrel subdomain of the mt-AlaRS editing domain and involve an arginine to tryptophan substitution, while a β-barrel subdomain variant (c.1616A>G, p.Tyr539Cys) has also been reported *in trans* with the p.Arg592Trp founder mutation (24). However, the mutated residues are not located in the editing core of mt-AlaRS. Previous *in silico* modelling showed that the mt-AlaRS Arg592 residue is surface exposed and forms one salt bridge with Glu567, which is invariant in this position among cytosolic, mitochondrial and bacterial homologues of the enzyme. The p.Arg592Trp founder mutation was predicted to have a deleterious effect on tRNA binding and a severe reduction in aminoacylation activity of mt-AlaRS but with no effect on editing activity or protein stability (22). Similarly, Arg580 is also solvent exposed but in contrast to Arg592, it forms a number of different non-covalent bonds with neighbouring residues that suggests importance of arginine in this position for protein stability (Fig. 3). Hence, substitution of the Arg580 residue with tryptophan is predicted to compromise mt-AlaRS protein folding and stability.

Indeed, quantification of mt-AlaRS protein levels confirmed a statistically significant decrease in fibroblasts, skeletal and cardiac muscle from both patients harbouring the p.Arg580Trp variant and in fibroblasts from two patients harbouring at least one copy of the p.Arg592Trp founder allele (Fig. 4A and B, Fig. 5A and B). Furthermore, mt-AlaRS protein levels appeared to be decreased more in fibroblasts from Patient 1, who harboured the p.Arg580Trp allele, compared to two patients with at least one copy of the p.Arg592Trp founder mutation. Hence, this supports our *in silico* modelling of the p.Arg580Trp missense change. Decreased mt-AlaRS protein levels were accompanied by severe multiple OXPHOS defects in patient skeletal and cardiac muscle (Fig. 5A) but were absent in patient fibroblasts (Fig. 4A). This suggests that skeletal and cardiac muscle are more susceptible to mitochondrial protein synthesis defects, while mt-AlaRS activity is essential in cardiac cells during early development. Our *in vitro* studies of mutant p.Arg580Trp mt-AlaRS also showed decreased protein stability when overexpressed in HEK293T cells (Fig. 6E). Decreased mt-tRNA^Ala^ levels were also observed in patient cardiac muscle (Fig. 5C), confirming a deleterious effect on mitochondrial protein synthesis. However, our *in vitro* data clearly showed that the p.Arg580Trp variant had little effect on amino acid activation and aminoacylation activities (Fig. 6), which was expected since it is not located near the aminoacylation active site. These data also showed that the p.Arg580Trp variant did not accumulate more mischarged tRNA^Ala^ in misaminoacylation when compared to wild-type human mt-AlaRS, since the residue is distant from the editing active site (∼40 Å). Moreover, it is also not in the potential tRNA entrance pathway into the editing active site during tRNA 3'-end translation. Taken together, our data suggest that the p.Arg580Trp variant impacts on stability of mt-AlaRS protein but not aminoacylation or editing activities and that the β-barrel subdomain has a critical role in protein folding and stability. The combination of the p.Arg580Trp variant *in trans* with a second, heterozygous loss-of-function allele as detected in our patients, suggests that marked loss of mt-AlaRS is sufficient to cause a severe defect of mitochondrial protein synthesis that manifests as fatal infantile cardiomyopathy. Although skeletal and cardiac muscle appear more susceptible to mitochondrial protein synthesis defects than other cell types such as fibroblasts, data suggest that specific loss of mt-AlaRS during early development has a highly detrimental effect on cardiac cells. By contrast, loss of other mt-aaRS does not affect cardiac function during early life. For example, a loss of mt-GluRS protein due to recessively inherited *EARS2* variants leads to a severe, lethal neonatal leukoencephalopathy with thalamus and brainstem involvement and high lactate (39), but without cardiac involvement. Our findings continue to support the hypothesis that there is an enhanced requirement for mitochondrial protein synthesis in the heart in early life and that a 50% reduction of aminoacylation activity is sufficient to maintain mitochondrial translation (22). On the other hand, recessively inherited variants causing other *AARS2*-related phenotypes are predicted to cause only a partial loss of mt-AlaRS protein level or aminoacylation activity, manifesting in childhood or adulthood phenotypes but not in the heart (22). However, steady-state mt-AlaRS protein levels have not yet been assessed in tissue from patients with other *AARS2*-related disorders. Furthermore, additional *in vitro* studies of the p.Arg592Trp founder mutation are necessary to confirm any impact on amino acid activation, aminoacylation and misaminoacylation activities. This would determine whether p.Arg580Trp and p.Arg592Trp have a shared biological mechanism or if disturbed tRNA binding with a severe loss of aminoacylation activities or marked instability of mt-AlaRS are two mechanisms that manifest clinically with fatal cardiomyopathy, concurring with *in silico* models.

Overall, our data strengthen the importance of mt-AlaRS in cardiac muscle during early embryonic development and the relationship between β-barrel subdomain variants (p.Arg580Trp and p.Arg592Trp) and the manifesting *AARS2*-related mitochondrial disease phenotypes.

## Materials and Methods

### Ethical compliance and informed consent

Informed consent for diagnostic and research-based studies was obtained for all subjects in accordance with the Declaration of Helsinki protocols and approved by local institutional review boards.

### Histopathology, biochemical and molecular studies

Diagnostic skeletal muscle biopsies from both patients, endo-myocardial biopsy and post-mortem cardiac muscle from Patient 2 were processed and mounted on glass slides according to standard procedures. Skeletal muscle biopsies (Patient 1 and 2) and post-mortem cardiac muscle (Patient 2) were subjected to cytochrome COX, SDH and sequential COX-SDH histochemical reactions (40). Mitochondrial OXPHOS activities (complexes I–IV) relative to citrate synthase (CS) were measured in skeletal (Patient 1 and 2) and cardiac muscle (Patient 2) homogenates as previously described (41). Whole mitochondrial genome sequencing of both patients was performed to exclude pathogenic variants. Quantitative real-time PCR assay of skeletal (Patient 1 and 2) and cardiac muscle DNA (Patient 2) was performed to assess mtDNA copy number, according to standard protocols.

### Next-generation sequencing and genetic investigations

WES, filtering and candidate variant analysis was performed for Patient 1 as described previously (39). In Patient 2, bidirectional sequence from WGS was prepared using the Kapa Hyper library prep omitting PCR, sequenced using the Illumina HiSeq 2500 system utilizing paired end 2 × 125 base pair reads with v4 Chemistry, aligned to reference gene sequences based on human genome build GRCh37/UCSC hg19 and variants were analysed using custom-developed software; RUNES and VIKING (42,43). WES was performed on unaffected parents of Patient 2. Patient 2 was sequenced to a depth of 111.52 Gb for a mean coverage of ∼37x. Variants were filtered with a MAF less than 1% and then prioritized by the American College of Medical Genetics categorization. Align GVGD (http://agvgd.hci.utah.edu/agvgd_input.php) (38), SIFT (http://sift.jcvi.org/) (37) and PolyPhen-2 (http://genetics.bwh.harvard.edu/pph2/) (36) were used to assess pathogenicity of missense variants. Identified candidate variants were confirmed by Sanger sequencing. Long-range PCR of the *AARS2* (NM_020745.2) gene was performed using forward (5′-GTGGGGTCAGCCCTGTTCCT-3′) and reverse (5′-CAGGAAGGCTGCCTCGTCCT-3′) primers.

### Structural modelling

Structure prediction for human mt-AlaRS with bound tRNA^Ala^ and alanyl-adenylate in the aminoacylation site was done as earlier described (22). Briefly, a multiple sequence alignment of different cytoplasmic, mitochondrial and bacterial homologues of human mt-AlaRS was done using Promals3D server. The resulting alignment was submitted to SWISS-MODEL server. As a template for human mt-AlaRS structure modelling, the full-length AlaRS from *Archaeoglobus fulgidus* was used (PDB id 3WQY chain A) (44). Docking of the tRNA and alanyl-adenylate into the model and structure analysis was done using Discovery Studio v4.5 (BioVia) software.

### Cell culture

Patient and control cultured skin fibroblasts were grown in minimum essential medium (MEM) (Gibco) or Dulbecco's Modified Eagle Medium supplemented with 10% foetal bovine serum (Gibco), 1× MEM vitamins (Sigma), 1× non-essential amino acids (Sigma), 50 U/ml penicillin, 50 μg/ml streptomycin (Sigma), 100 mm sodium pyruvate solution (Sigma), 0.05 mg/ml uridine aqueous solution and 2 mm L-glutamine (Sigma).

### Western blot analysis

Fibroblast lysates (50 μg), skeletal (25–50 μg) and cardiac muscle (15–50 μg) homogenates were separated by 12% SDS-PAGE and electrophoretically transferred to polyvinylidene difluoride (PVDF) membranes (Bio-Rad). Primary antibodies used were specific to mt-AlaRS (ab197367, Abcam), NDUFB8 (ab110242, Abcam), SDHA (ab14715, Abcam), UQCRC2 (ab14745, Abcam), MT-COI (ab14705, Abcam), ATP5B (ab14730, Abcam), β-actin (A5316, Sigma) and VDAC (ab14734, Abcam). Following incubation with horseradish peroxidase-conjugated secondary antibodies (Dako) for 1 h at room temperature, detected proteins were visualized with Clarity Western ECL substrate (Bio-Rad) using the Bio-Rad ChemiDoc MP with Image Lab software according to manufacturer’s guidelines.

### Northern blotting

Total RNA was extracted from cultured fibroblasts and cardiac muscle using Trizol reagent (ThermoFisher Scientific) according to the manufacturer’s instructions. To preserve the aminoacylation state the final RNA pellet was re-suspended in 10 mm NaOAc at pH 5.0. To investigate the aminoacylation status of mt-tRNAs, RNA (4 μg) was separated on long (16 cm length) 6.5% polyacrylamide gel (19:1 acrylamide:bis-acrylamide) containing 8 M urea in 0.1 M NaOAc, pH 5.0. The control of fully deacylated tRNA (dAc) was obtained by incubation of control RNA at 75°C (pH 9.0) for 15 min. To determine mt-tRNA^Ala^ steady-state levels the samples were run on 10 cm gel. Northern hybridization was performed with ϒ-32P labelled oligonucleotide probes: 5′-GTGGCTGATTTGCGTTCAGT-3′ for the mt-tRNA^Ala^, 5′-GAGTCGAAATCATTCGTTTTG-3′ for the mt-tRNA^Arg^ and 5′-GTTGTTAGACATGGGGGCAT-3′ for mt-tRNA^Ser(AGY)^. Radioactive signal was detected by PhosphorImager plate using Typhoon scanner and quantified with the ImageQuant v5.0 software (GE Healthcare).

### Cloning, gene expression and protein purification of human mt-AlaRS

Gene expression and protein purification of human mt-AlaRS and the p.Arg580Trp mutant were performed as earlier described (33). The host strain used was *Escherichia coli* Rosetta (DE3).

### Transcription of human mt-tRNA^Ala^

Transcription of human mt-tRNA^Ala^ and ^32^P-labelling of human mt-tRNA^Ala^ were performed as earlier described (33).

### ATP-PPi exchange assay

ATP-PPi exchange assay of human mt-AlaRS and the p.Arg580Trp mutant was performed as earlier described (33). The reaction buffer contained 50 mm Tris-HCl (pH 8.0), 20 mm KCl, 10 mm MgCl_2_, 2 mm DTT, 4 mm ATP, 5 mm Ala, 2 mm tetrasodium [^32^P]pyrophosphate and 200 nM enzyme at 37°C.

### Aminoacylation activity

Aminoacylation assay of human mt-tRNA^Ala^ and the p.Arg580Trp mutant was performed as earlier described (33). The reaction buffer contained 50 mm Tris-HCl (pH 8.0), 20 mm KCl, 10 mm MgCl_2_, 2 mm DTT, 4 mm ATP, 1 M Ser, 4 μM human mt-tRNA^Ala^, 0.455 μM [^32^P]tRNA^Ala^ (54 000 cpm) and 2 μM human mt-AlaRS and the p.Arg580Trp mutant at 37°C.

### Misaminoacylation assay

Misaminoacylation assay was performed in a reaction buffer containing 50 mm Tris-HCl (pH 8.0), 20 mm KCl, 10 mm MgCl_2_, 2 mm DTT, 4 mm ATP, 1 M Ser, 4 μM human mt-tRNA^Ala^, 0.455 μM [^32^P]tRNA^Ala^ (54 000 cpm) and 2 μM human mt-AlaRS and the p.Arg580Trp mutant at 37°C. Assay processing was performed as previously described (45). The amount of Ser-[^32^P]AMP produced was calculated by multiplying the total amount of tRNA^Ala^ (including [^32^P]tRNA^Ala^ and unlabelled tRNA^Ala^) by the relative level of charged tRNA^Ala^ in the aliquots: [Ser-[^32^P]AMP/(Ser-[^32^P]AMP + [^32^P]AMP)].
